# HPLC Analysis and Biochemical Characterization of LOX from *Eschscholtzia californica* Cham.

**DOI:** 10.3390/molecules22111899

**Published:** 2017-11-04

**Authors:** Renáta Kollárová, Ivana Holková, Drahomíra Rauová, Barbora Bálintová, Peter Mikuš, Marek Obložinský

**Affiliations:** 1 Department of Cell and Molecular Biology of Drugs, Faculty of Pharmacy, Comenius University in Bratislava, Kalinčiakova 8, 832 32 Bratislava, Slovak Republic; holkova@fpharm.uniba.sk (I.H.); balintova@fpharm.uniba.sk (B.B.); oblozinsky@fpharm.uniba.sk (M.O.); 2 Department of Pharmaceutical Analysis and Nuclear Pharmacy, Faculty of Pharmacy, Comenius University in Bratislava, Odbojárov 10, 832 32 Bratislava, Slovak Republic; rauova.drahomira@fpharm.uniba.sk (D.R.); mikus@fpharm.uniba.sk (P.M.); 3 Toxicological and Antidoping Center, Faculty of Pharmacy, Comenius University in Bratislava, Odbojárov 10, 832 32 Bratislava, Slovak Republic

**Keywords:** lipoxygenase, hydroxy fatty acid isomers, *Eschscholtzia californica* Cham., purification, HPLC analysis, biochemical parameters

## Abstract

Background: Plant lipoxygenases (LOXs, EC 1.13.11.12) are involved in lipid degradation, regulation of growth and development, senescence, and defence reactions. LOX represents the starting enzyme of the octadecanoid pathway. The aim of the work was to purify LOX from California poppy (*Eschscholtzia californica* Cham.), to determine its biochemical properties and to identify and quantify the products of LOX reaction with unsaturated fatty acids. Methods: LOX from California poppy seedlings was purified by hydrophobic chromatography (Phenyl-Sepharose CL-4B) and by ion-exchange chromatography (Q-Sepharose). The isolated LOX was incubated with linoleic acid used as a substrate. The HPLC experiments were performed with the Agilent Technologies 1050 series HPLC system. For the preparative separation of a mixture of hydroxy fatty acids from the sample matrix, the RP-HPLC method was used (column 120-5 Nucleosil C18). Then, the NP-HPLC analysis (separation, identification, and determination) of hydroxy fatty acid isomers was carried out on a Zorbax Rx-SIL column. Results: The purified LOX indicates the presence of a nontraditional plant enzyme with dual positional specificity (a ratio of 9- and 13-hydroperoxide products 1:1), a relative molecular mass of 85 kDa, a pH optimum of 6.5, an increasing activity stimulation by CaCl_2_ till 2 mM, and a high substrate reactivity to linoleic acid with kinetic values of K_M_ 2.6 mM and V_max_ 3.14 μM/min/mg. Conclusions: For the first time, the LOX from California poppy seedlings was partially purified and the biochemical properties of the enzyme were analyzed. A dual positional specificity of the LOX found from California poppy seedlings is in agreement with the results obtained for LOXs isolated from other Papaveraceaes. A 1:1 ratio of 9-/13-HODE is attractive for the simultaneous investigation of both biotic stress responses (indicated by the 9-HODE marker) and the biosynthesis of jasmonic acid and jasmonates (indicated by the 13-HODE marker).

## 1. Introduction

California poppy (*Eschscholtzia californica* Cham.) of the Papaveraceae family is an annual plant originating from California [1]. The entire plant produces a mixture of tertiary and quaternary isoquinoline alkaloids. In traditional medicine, it is used for its sedative, anxiolytic, and spasmolytic effects. Among the alkaloids present in California poppy, sanguinarine has attracted increasing attention. This benzophenanthridine alkaloid has antimicrobial activity and plays a role in plant defence [2]. Besides its antimicrobial properties, sanguinarine also exhibits antiviral and cytotoxic activities [3].

Lipoxygenases (LOXs, linoleate:oxygen oxidoreductases, EC 1.13.11.12) are a class of widespread dioxygenases that catalyze the regio- and stereo-specific addition of molecular oxygen to polyunsaturated fatty acids (PUFA) containing one or more *cis*,*cis*-1,4-pentadiene systems of double bonds to form conjugated mono-hydroperoxy fatty acids. Linoleic (LA) and linolenic acids (LeA) are the most common substrates for LOXs in plants [4,5]. In higher plants, the oxygenation of LA by a LOX enzyme produces the corresponding 9- or 13-hydroperoxide derivatives (9- or 13-HPODE). According to the positional specificity of LA dioxygenation, plant LOXs are classified as 13-LOX or 9-LOX. The enzyme is characterized as 13-LOX when the product formed is 13-hydroperoxy-9(*Z*),11(*E*)-octadecadienoic acid (13-HPODE) and 9-LOX when 9-hydroperoxy-10(*E*),12(*Z*)-octadecadienoic acid (9-HPODE) is predominantly produced [4,6]. Recently, LOX isoenzymes with dual positional specificity and producing both HPODE isomers have been reported [7,8,9].

Plants usually express several LOX isoforms. The intracellular location of LOXs may be cytosolic or organelle-associated. LOXs are present in the majority of plant organs, and their gene expression changes during developmental stages and is regulated by different forms of stress, such as wounding or pathogen attack [10].

LOXs are enzymes implicated in plant lipid metabolism. It has been proposed that these enzymes play an important role in the formation of signalling molecules, such as jasmonic acid and phytodienoic acid, at distinct stages of development and in plant defence responses [10,11]. LOX catalyzes the first reaction in the pathway, collectively named the lipoxygenase (octadecanoid) pathway, containing at least seven different branches. The key intermediates of this pathway (formed by the LOX) are hydroperoxides of fatty acids that are transformed into their corresponding metabolites called oxylipins [12]. The 9-hydroperoxides are enzymatically converted into a variety of oxylipins in plants, which are major compounds of the flavours and aromas of many fruits. The 9-LOXs also have a role in biotic stress responses. On the other hand, only 13-LOXs activate the biosynthesis of jasmonates and jasmonic acid, typically known as a wounding hormone [13]. Moreover, 13-LOX mediates the mobilization and release of 13-HODE during germination [14]. Therefore, the positional specificity of LOX is the crucial factor in determining whether the LOX is involved in the biosynthesis of jasmonic acid or not. Oxylipins generated by the LOX pathway have important biological activities as signalling molecules such as jasmonates, antimicrobial and antifungal compounds such as leaf aldehydes or divinyl ethers, and volatile compounds such as leaf alcohols [15,16].

Although California poppy is an interesting model object for the study of secondary metabolite production (sanguinarine), there have been no references about the biochemical properties of California poppy LOX before this study. The highest activity of LOX was observed in newly developing tissues [10]. Therefore, the activity of the LOX during the germination of California poppy seeds was analyzed in the present work. A characterization of the LOX from California poppy with respect to its biochemical properties could help to broaden our knowledge about LOXs in higher plants.

The main goal of the work was to isolate and purify the LOX from California poppy seedlings (*Eschscholtzia californica* Cham.) and to determine its biochemical properties, such as substrate and positional specificity, pH optimum, the effect of calcium ion concentration, relative molecular mass, and kinetic parameters.

## 2. Results and Discussion

### 2.1. LOX Activity during Germination of California Poppy Seeds

**Figure 1:**
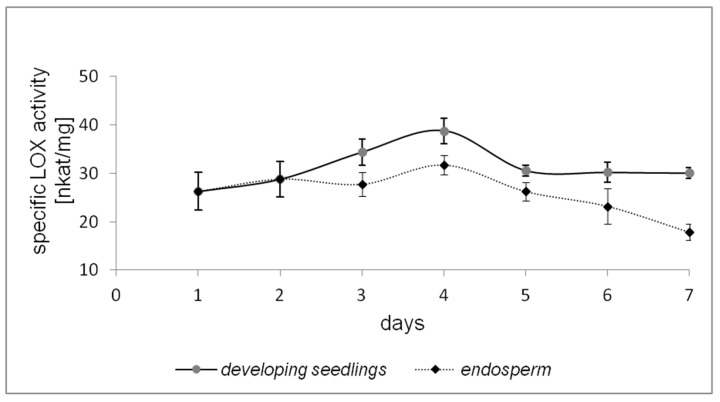
Changes of specific lipoxygenase (LOX) activity in endosperm and in developing seedlings (1st–7th day after germination). Linoleic acid was used as a substrate. Values are means ± SD from triplicate experiments.

Changes in LOX activities during the germination of California poppy were analyzed in 1–7 day(s)-old seedlings (Figure 1). The enzyme activity increased with the period of germination, with the maximum on the 4th day after germination, then the LOX activity decreased. Higher LOX activities were determined in developing seedlings than in endosperm. The maximum specific activity of LOX was detected in 4-days-old developing seedlings without endosperm (38.77 ± 2.59 nkat/mg). Based on these results, 4-days-old seedlings without endosperm were chosen for the purification of California poppy LOX.

Oilseed germination is characterized by the mobilization of storage lipids, which provide energy and serve as a major carbon source for the growth of seedlings. It is well-known that during germination, new LOXs are synthetized in the seedlings and cotyledons, with a maximal accumulation of LOX protein from a few hours to few days after germination. This corresponds with results observed in sunflower seedlings (*Helianthus annuus* L.) during germination [17]. The LOX activity increased from 1 to 5 days of germination, and then the enzyme activity declined on the 6th day. The highest LOX activity was reported in 5-days-old sunflower seedlings. Similarly, purified LOX isolated from opium poppy seedlings showed the highest activity on the 4th day after germination [9]. Terp et al. [18] monitored the formation of oxylipins, which are indicative of endogenous LOX activity during the first 2 weeks of germination, in oilseed rape (*Brassica napus* L.). In the case of seeds germinating in the dark, the maximal accumulation of oxylipins was observed on the 3rd day of germination. All of these results suggest that high LOX levels found during germination indicate the role of this enzyme in plant growth and development.

### 2.2. Purification of California Poppy LOX

**Figure 2:**
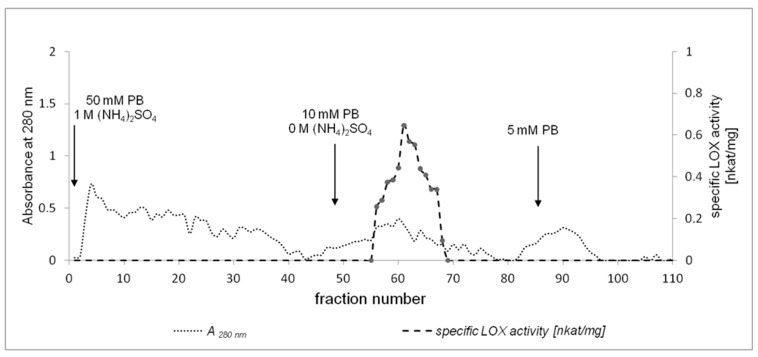
Elution profile of California poppy LOX on a Phenyl-Sepharose CL-4B column. The column was equilibrated with 50 mM phosphate buffer (PB) with 1 mM ammonium sulfate and eluted with 10 mM and 5 mM phosphate buffer. Proteins were measured at 280 nm and LOX activity at 234 nm.

**Figure 3:**
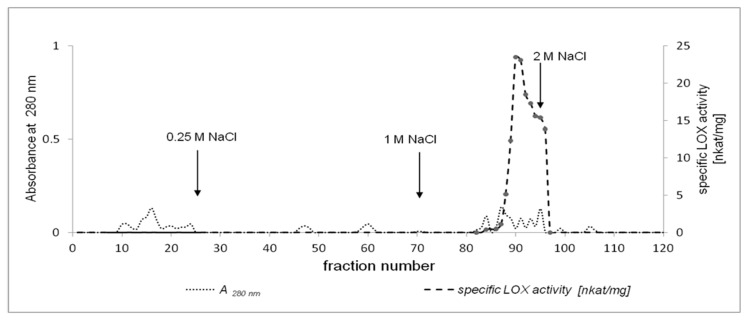
Elution profile of California poppy LOX on a Q-Sepharose column. The column was equilibrated with 100 mM phosphate buffer and eluted with 100 mM phosphate buffer with increasing concentration of NaCl (0.25 M, 1 M, and 2 M NaCl). Proteins were measured at 280 nm and LOX activity at 234 nm.

LOX purification consisted of several steps, as summarized in Table 1. Acetone powder was found to be necessary for the optimization of LOX extraction from California poppy seeds, the preservation of enzyme activity, the delipidation of plant material, and removing other impurities. Some undesirable impurities that might act as inhibitors of the LOX may also have been removed. Acetone powder was prepared from 4-days-old California poppy seedlings without endosperm. Solubilised acetone powder was centrifuged at 12,000× *g* and the supernatant was used as a crude extract for the purification of the LOX from California poppy. The crude extract was subjected to ammonium sulfate precipitation to 60% saturation as a first step of LOX purification. The specific activity of the LOX after precipitation was 0.34 nkat/mg. Hydrophobic chromatography on a Phenyl-Sepharose CL-4B column was selected as a subsequent stage for the partial purification process. As shown in Figure 2, one major peak was detected with LOX activity (0.49 nkat/mg) after hydrophobic chromatography. Partially purified LOX was further purified by ion-exchange chromatography on a Q-Sepharose column (Figure 3). An overall 69-fold purification was achieved. The specific activity of the pure LOX reached 23.47 nkat/mg when LA was used as a substrate.

Williams and Harwood [19] isolated the LOX from callus cultures of *Olea europaea* L. In this experiment, acetone powder from the cultures was used to prevent the loss of LOX activity. Ammonium sulfate precipitation up to 60% saturation achieved a 12-fold purification of the LOX. After subsequent ion-exchange chromatography on a DEAE Sephadex A50, two LOX isoenzymes with 48 and 55 purification factors were obtained. The results of our studies correspond well with those obtained by Holková et al. [9], where also one LOX isoenzyme from opium poppy was indicated. Vanko et al. [20] isolated and purified the LOX from opium poppy chloroplasts with a purification fold of 126.1 by a combination of ion-exchange chromatography and affinity chromatography. Other comparable results to ours were reported by Lorenzi et al. [21]. Differential centrifugation and hydrophobic chromatography yielded a 65-fold purified LOX isolated from fruit of the olive tree (*Olea europaea* L.). Two LOX isoforms were isolated from broad beans (*Vicia faba* L.) [22], and a 327-fold purified LOX was isolated from banana leaves by ammonium sulfate fractionation, hydroxyapatite column separation, and gel filtration [23].

### 2.3. Determination of the Relative Molecular Mass of the LOX

**Figure 4:**
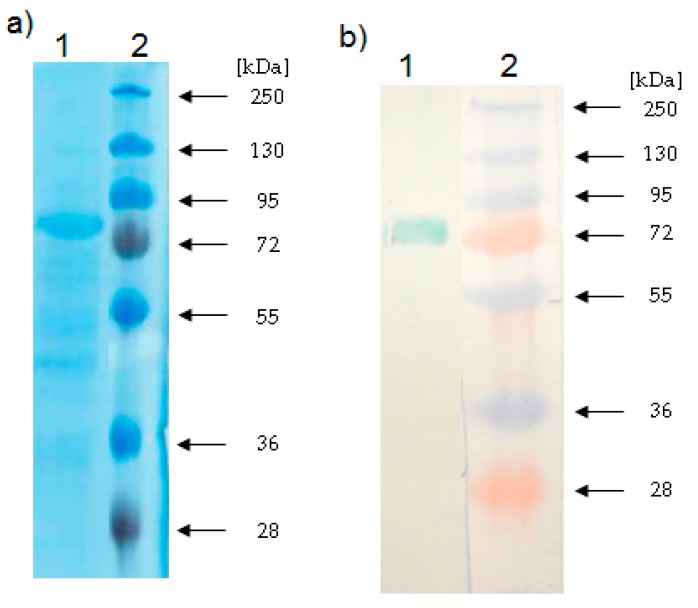
SDS-PAGE and Western blot analysis of the purified California poppy LOX (**a**) SDS-PAGE (10% acrylamide gel stained with Coomasse Brilliant Blue) of purified LOX from California poppy. (**b**) Western blot analysis of a gel with the same samples. Lane 1: purified LOX from California poppy after ion-exchange chromatography. Lane 2: protein marker PageRulerTM Plus Prestained Protein Ladder (Fermentas, Lithuania) (11–250 kDa).

The purity of California poppy LOX was detected by SDS-PAGE in 10% polyacrylamide gel. The purified LOX appeared as a unique band at 85 kDa (Figure 4a) in Coomasse Brilliant Blue stained gel. After an immunoblot with anti-soybean LOX antibodies, one band showing the same molecular mass was identified (Figure 4b). In general, plant LOXs have a relative molecular mass in the range of 94–104 kDa [24]. However, in some plant sources, LOXs with lower molecular mass have also been found. Our results are in good agreement with previously published data for some plant LOXs, such as in banana leaves [23] and in common bean seeds [25]. The LOX 1 isoform isolated from pearl millet has an Mr of approximately 85 kDa [26]. Interestingly, the Mr of the LOX from opium poppy seedlings is 78 kDa [9] and the LOX isoenzyme isolated from the chloroplast of opium poppy leaves has an Mr of 92 kDa [20].

### 2.4. Optimal pH for LOX Activity

**Figure 5:**
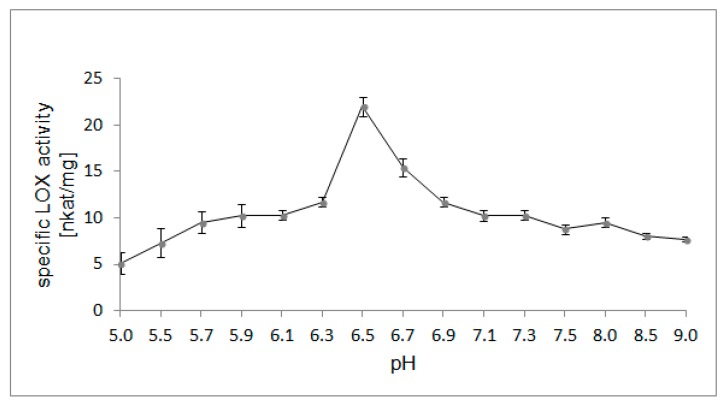
Effect of pH on the activity of the purified LOX from California poppy seedlings. Linoleic acid was used as a substrate. The buffer system included 100 mM potassium phosphate buffer ranging from pH 5.0 to 9.0. Values are means ± SD from triplicate experiments.

The enzymatic activity was analyzed spectrophotometrically at different pH values (range 5.0–9.0) using LA as a substrate. A pH of 6.5 was determined to be the optimal pH for the activity of purified LOX from California poppy (Figure 5). At a pH of 8.5, 37% of maximum activity was observed, and at a pH of 5.0, only 23% of maximum activity was observed. The optimum pH of the LOX enzyme from other plants has been estimated to be between a wide pH range (5.5–9.5). On comparison to the pH optimum for LOXs isolated from other plant sources, the same pH optimum for LOX activity was reported for the enzyme from opium poppy seedlings [9], avocado fruit [27], tomato [28], and pea roots [29]. Optimum pH is also known to be related to the type of LOX isoenzyme. Soybean LOX-1 has a pH optimum at 9.0 and LOX-2 isoform at 6.5 [30]. The effect of pH on LOX activity may be explained by the fact that at different pH the catalytic site of the enzyme has different conformations and iron oxidation states [31].

### 2.5. HPLC Analysis of LOX Reaction Products

LOXs catalyze the oxygenation of PUFAs with a *cis*,*cis*-1,4-pentadiene system to produce corresponding conjugated diene hydroperoxides. Plant LOXs are usually classified as 9-LOX or 13-LOX on the basis of their product specificity. To investigate the positional specificity of the purified LOX, the HPLC experiment was performed in two steps.

**Figure 6:**
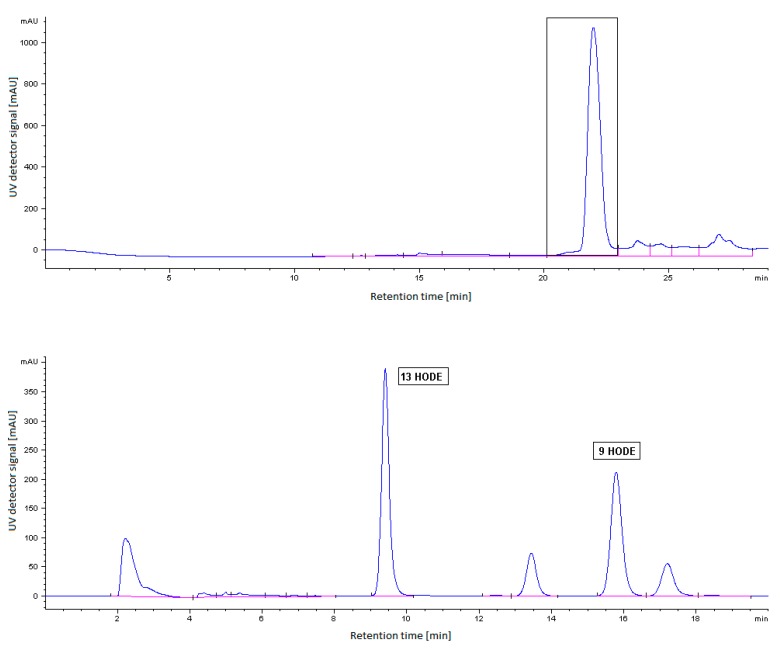
HPLC analysis of LOX reaction products for the determination of the positional specificity of California poppy LOX. Upper panel: Chromatographic profile of the preparative RP-HPLC separation of the sample obtained from the incubation of the isolated LOX with Linoleic acid (LA) as a substrate. A marked peak represents an isolated fraction used for the subsequent NP-HPLC separation of HODEs. The eluate of the isolated fraction was collected in the interval of 20–23 min. The elution time of the marked peak is 21.971 min. Lower panel: Chromatographic profile of the fine NP-HPLC separation of 9-/13-HODE products present in the isolated RP-HPLC fraction. Two unidentified abundant peaks in the NP-HPLC profile could be structurally related compounds, such as fatty acids originating from the plant matrix (based on their similar elution and UV absorbance properties). The elution times of 13-HODE and 9-HODE are 9.398 and 15.778 min, respectively. For the RP- and NP-HPLC analytical conditions see Section 3.5.

In the first step, the RP-HPLC preparative separation of the sample obtained from the incubation of the isolated LOX with LA as a substrate was carried out according to the procedure described in [20]. The reaction products, hydroperoxy fatty acids, were reduced with NaBH_4_ to the corresponding hydroxy fatty acid and then separated from the incubated sample matrix. The reversed-phase effectively removed a majority of the interfering residual sample matrix constituents present in the isolated LOX employed for the incubation experiment, as can be seen in Figure 6 (upper chromatographic profile). The mixed peak absorbing at 234 nm in the RP-HPLC profile containing the hydroxy fatty acids (i.e., LA products as well as other possible structurally related compounds originating from the plant matrix) was isolated and collected.

In the second step, the mixed peak fraction was subjected to an NP-HPLC analysis of each individual 9- and 13-HODE: see the lower chromatographic profile in Figure 6. The normal-phase HPLC mode was optimal for the fine separation of hydroxy fatty acids isomers, i.e., distinguishing among structurally related products including 13-HODE, 9-HODE, and other possible structurally related fatty acids originating from the plant matrix (the other two abundant peaks in the NP-HPLC profile of Figure 6). For identification, the retention times of the reaction products of the California poppy LOX were compared with the reaction products of soybean LOX and authentic standards of 9- and 13-HODE. Based on the calibration curves prepared using 9-HODE and 13-HODE standards, the amount of LOX products formed during the reaction with LA was determined.

The HPLC analysis of the purified California poppy LOX products showed a dual positional specificity of the enzyme (lower chromatographic profile in Figure 6) with the ratio of 9-/13-HODE being about 1:1 when LA was used as a substrate at a pH of 6.5. The concentration of 9-HODE in the incubation mixture was calculated to be 3.86 ± 0.76 μg/mL and that of 13-HODE was 4.30 ± 0.60 μg/mL. For a comparison, commercial soybean LOX was analyzed. The main reaction product of soybean LOX was 13-HODE (77%), while 9-HODE was formed at 23%. Interestingly, plant LOXs with dual positional specificity are classified as nontraditional LOX enzymes able to synthetize compounds that play a role in developmental processes and defence responses in plants. Dual-specific LOX are supposed to be able to supply hydroperoxides both for jasmonate and plant leafy volatiles biosynthesis [20]. The fact that California poppy LOX has a dual positional specificity with the ratio of 9-/13-HODE being about 1:1 is in agreement with results obtained for LOXs isolated from other Papaveraceaes, such us opium poppy seedlings [9] and chloroplast poppy LOX [20]. LOXs that produce both 9-HODE and 13-HODE were detected in olive fruit (with the ratio of 9-/13-HPODE being 2:1) [32], and nontraditional dual-specific LOX was expressed in maize seedlings in response to wounding and stress [33].

### 2.6. Substrate Specificity of the LOX

**Figure 7:**
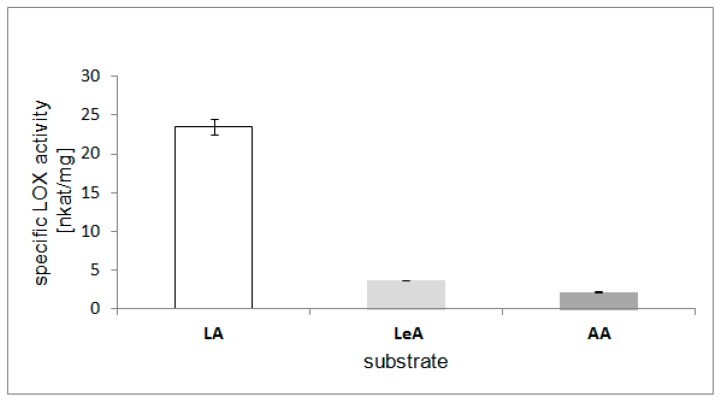
Substrate specificity of purified LOX from California poppy seedlings. Linoleic acid (LA), linolenic acid (LeA), and arachidonic acid (AA) were used as substrates. Values are means ± SD from triplicate experiments.

The substrate specificity of the purified LOX was investigated using LA, LeA, and arachidonic acid (AA) as substrates (Figure 7). The highest relative enzyme activity was obtained with LA (100%), followed by LeA (16%) and AA (9%). These findings correspond with the features found for other plant LOXs, which showed the highest activity when the reaction substrates were C18 PUFA, such as LA or LeA. Furthermore, in many oilseeds plants (sunflower, cucumber), LA is a major fatty acid esterified in storage triacylglycerols [14]. According to this, LA may be considered to be the main substrate for the LOX in oilseed plants. LOXs from other plants have also shown high preference to LA [34,35]. Interestingly, the eggplant LOX showed high affinity to LA but did not metabolize AA [35]. A LOX isolated from pea (*Pisum sativum* L.) showed a substrate preference to LA, followed by AA and LeA [36].

### 2.7. Stimulatory Effect of Ca^2+^ Ions on LOX Activity

**Figure 8:**
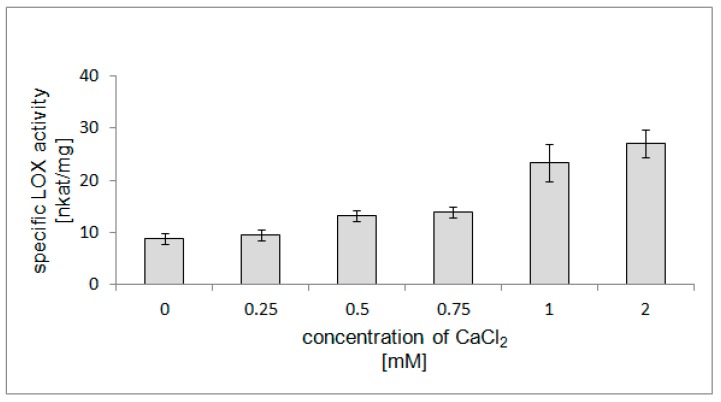
The effect of Ca^2+^ concentration on the activity of the purified LOX from California poppy seedlings. Linoleic acid was used as a substrate. Values are means ± SD from triplicate experiments.

The activity of several plant LOX isoenzymes is calcium dependent, and therefore the effect of Ca^2+^ concentration on the activity of the purified LOX was evaluated. To examine the Ca^2+^ dependency of the purified LOX from California poppy, the enzyme activity was analyzed spectrophotometrically at a pH of 6.5. Calcium ions markedly increased LOX activity, and the results indicated that the maximal stimulation of enzyme activity to 3.1 fold was observed at a concentration of 2 mM of CaCl_2_ (Figure 8). The ability of calcium to stimulate LOX activity has been reported in avocado fruit [27] and in opium poppy seedlings, in which the highest LOX activity was observed at 0.75 mM Ca^2+^ concentration [9]. In plants, calcium is known to play roles in senescence and defence against pathogens, and a possible role for calcium in the oxidation of PUFA has been proposed. Although LOX activity was thought to be calcium-independent in plants, the ability of calcium to stimulate LOX activity has been reported in some plants. Furthermore, calcium may regulate the LOX-catalyzed oxidation of PUFA by more than one mechanism [37].

### 2.8. Kinetic Paramaters

On the basis of the Lineweaver–Burk method, the kinetic parameters of the purified LOX from California poppy were determined using LA as a substrate. The value of the Michaelis–Menten constant of the purified LOX was 2.6 mM and the V_max_ was 3.14 μM/min/mg. Also, the kinetic parameters in the presence of LeA as a substrate were analyzed (data not shown). Our results correspond well to those obtained for the LOX from green pea (K_M_ 2.33 mM) [38] and banana leaves (V_max_ 2.4 μM/min/mg) [23] using LA as a substrate. Similar results for V_max_ were reported for the LOX purified from eggplant (2.2 μM/min/mg) [35] and the LOX from *Cichorium intybus* L. (2.05 μM/min/mg) [34]. For the tomato LOX, the value of K_M_ was 4.2 mM [39] and for chloroplast opium poppy LOX 1.78 mM [20]. Lower K_M_ values obtained towards LA were for the olive LOX (0.89 mM) [27] and the membrane-bound LOX isolated from banana leaves (0.15 mM) [23].

## 3. Materials and Methods

### 3.1. Plant Material

California poppy seeds (*Eschscholtzia californica* Cham., Papaveraceae) were obtained from SEVA SEED, Valtice, Czech Republic. Seeds were treated using 5% sodium hypochlorite and rinsed with sterile water several times. Sterile seeds were sown on moistened cotton wool placed in Petri dishes and germinated in the dark at 25 °C and 70–80% relative humidity in an incubator (BINDER BD 115, Tuttlingen, Germany).

### 3.2. Preparation of Crude Extracts

After different periods of germination (1st–7th day), California poppy seedlings were harvested and separated from endosperm. Two grams (2 g) of plant material (endosperm or developing seedlings without endosperm) were homogenized in a precooled mortar using 10 mL of potassium phosphate buffer (100 mM, pH 6.5). Homogenates were centrifuged for 10 min at 12,000× *g* and 4 °C (Sigma 3-30K, Osterode am Harz, Germany). Then, the supernatant was centrifuged for 5 min under the same conditions and used for LOX activity determination.

### 3.3. Enzyme Purification

First, 54 g of 4-days-old developing seedlings without endosperm were ground with dry ice as described by Obložinský et al. [40]. Acetone powder (3.0 g) was homogenized at a low temperature with 55 mL of extraction buffer containing 25 mM potassium phosphate buffer (pH 6.0), 1 mM cysteine hydrochloride monohydrate, 0.5 mM ethylenediaminetetraacetic acid (EDTA), 10 mM sodium thiosulfate, and 1 mM phenylmethylsulfonyl fluoride (PMSF). The homogenate was filtered through a layer of cheesecloth and centrifuged at 12,000× *g* for 15 min at 4 °C.

All of the extraction and purification procedures were performed at 4 °C. The proteins in the supernatant fluid were precipitated to 60% (*w*/*v*) saturation with ammonium sulfate and centrifuged at 15,000× *g* for 30 min. The concentrated protein sample was dissolved in 3 mL of potassium phosphate buffer (50 mM, pH 7.0) containing 1 M ammonium sulfate and applied to a Phenyl-Sepharose CL-4B column (Ø 1.5 × 15 cm, Sigma-Aldrich, St. Louis, MO, USA) equilibrated with the same buffer. The resin was washed with 50 mM potassium phosphate buffer (pH 7.0) containing 1 M ammonium sulfate, and bound proteins were eluted with 10 mM potassium phosphate buffer (pH 6.5) containing 0.5 mM glutathione and 0.04% (*v*/*v*) Tween 20, followed by 5 mM potassium phosphate buffer (pH 6.5) containing 0.5 mM glutathione and 0.08% (*v*/*v*) Tween 20. Fractions of 2 mL were collected using an Econo-Column^®^ Pump (BIO-RAD, Hercules, CA, USA), and the protein elution profile was measured spectrophotometrically at 280 nm (Epoch Microplate spectrophotometer, BioTek Instruments, Winooski, VT, USA). Then, LOX activity was determined at 234 nm.

Pooled fractions with the highest LOX activity were concentrated to the final volume of 3 mL using 50 kDa membrane filter Amicon Ultra Centrifugal Filters Ultracel^®^-50K (Millipore, Massachusetts, USA) using centrifugation at 15,000× *g* for 15 min and at 4 °C. The sample was applied to a Q-Sepharose^®^ Fast Flow column (Ø 2 × 15 cm; Sigma-Aldrich, St. Louis, MO, USA) pre-equilibrated with potassium phosphate buffer (100 mM, pH 7.0). Elution was achieved with a discontinuous salt gradient of 0.25‒2.0 M NaCl. Two milliliter (2 mL) fractions were collected, and the absorbance at 280 nm was determined. Fractions with significant LOX activity were pooled, concentrated as described in the step above, and used for other analyses. The purified LOX was stored at −20 °C.

### 3.4. Lipoxygenase Activity Assay and Protein Determination

The lipoxygenase activity assay was performed spectrophotometrically (Epoch, Microplate spectrophotometer, BioTek Instruments, Winooski, VT, USA) at room temperature by measuring the increase of absorbance at 234 nm. The enzyme substrate, LA, was prepared according to Chen and Whitaker [41] and used in subsequent experimental stages. The reaction mixture contained 184 µL of potassium phosphate buffer (100 mM, pH 6.5), 21–25.1 µL of substrate solution (10 mM), and 0.9–5.0 µL of LOX enzyme preparation (according to protein concentration). Enzyme activity was expressed in katals. The protein content was determined according to the Bradford assay [42] with bovine serum albumin (Sigma-Aldrich, St. Louis, MO, USA) as a standard.

### 3.5. HPLC Analysis of LOX Positional Specificity

The HPLC analysis was carried out using Agilent Technologies HP 1050 Series LC (Waldbronn, Germany) coupled with a UV detector as described by Andreou et al. [43]. One hundred microliters (100 μL) of partially purified LOX was mixed with 900 μL of potassium phosphate buffer (100 mM, pH 6.5) and incubated for 30 min at 25 °C with 10 μL of substrate (10% methanol solution of LA, *v*/*v*). The reaction was stopped by adding 10 mg of NaBH_4_ and 100 μL of concentrated HCl. Hydroxyoctadecadienoic acids (HODEs) were isolated by diethyl ether (2 × 1.0 mL) and evaporated to dryness under a stream of nitrogen. The sample was dissolved in 100 μL of methanol and stored at −20 °C until analysis. After removing the organic solvent, the residue was reconstituted in 200 μL of methanol/water/acetic acid (85:15:0.1, *v*/*v*/*v*). The reaction products were then analyzed by reverse-phase HPLC (RP-HPLC) using a Nucleosil 120-5 C18 120 Å column (250 × 4 mm; Watrex, Praha, Czech Republic) eluted with a solvent system of: solvent A (methanol/acetic acid (100:0.1, *v*/*v*) and solvent B (deionized water) at a flow rate of 0.2 mL/min. The program of elution was as follows: 10 min with solvent system of 85% A and 15% B at a flow rate of 0.2 mL/min; 12 min with 100% A, flow rate 0.4 mL/min; 22 min with 100% A, flow rate 0.4 mL/min; 25 min with 85% A and 15% B, flow rate 0.4 mL/min; and 27 min with 85% A and 15% B, flow rate 0.4 mL/min. The eluate containing hydroxy fatty acids (peak fraction at 234 nm) was collected and evaporated to dryness under a stream of nitrogen. The residue was dissolved in 150 μL of hexane and aliquots of 50 μL were analyzed by normal-phase HPLC (NP-HPLC). The NP-HPLC of the hydroxy fatty acid isomers was carried out on a Zorbax Rx-SIL column (150 × 2.1 mm, 5 μm particle size, Agilent Technologies, Waldbronn, Germany) eluted with a solvent system of hexane/2-propanol/acetic acid (100:1:0.1, *v*/*v*/*v*) at a flow rate of 0.2 mL/min. The absorbance at 234 nm (conjugated diene system of the hydroxyl fatty acids) was set for all of the HPLC experiments. The products of the LOX reaction were identified using the authentic standards 9(*S*)-hydroxy-(10*E*,12*Z*)-octadecadienoic acid (9-HODE, 5 µg/mL) and 13(*S*)-hydroxy-(9*Z*,11*E*)-octadecadienoic acid (13-HODE, 5 µg/mL) (Cayman Pharma, Neratovice, Czech Republic) and their mixture containing the same concentration of both standards. For quantification of the LOX products in the NP-HPLC profiles, calibration curves for 9- and 13-HODE were obtained in the range 0.84–100.0 µg/mL and 1.29–100.0 µg/mL, respectively.

### 3.6. Effect of pH and Ca^2+^ Ions on LOX Activity

For determination of optimum pH on LOX activity, the spectrophotometric method was used with LA as a substrate. The enzyme activity was measured in 100 mM potassium phosphate buffer in the pH range of 5.0–9.0. LOX activity was evaluated at different CaCl_2_ concentrations in the range of 0.25–2.0 mM.

### 3.7. Kinetic Assay of Purified LOX

The K_M_ (Michaelis–Menten constant) and V_max_ (maximum velocity) values of the purified enzyme were estimated at different substrate concentrations (0.476–1.33 mM of LA as substrate) while keeping the concentration of the enzyme constant. Both parameters were calculated from the Lineweaver–Burk linearization plots relating 1/V to 1/[S] [44].

### 3.8. Substrate Specificity

To determine the substrate specificity of the purified LOX, different substrates (LA, LeA, and AA) were added to the reaction mixture at a concentration of 10 mM and LOX activity was measured spectrophotometrically at 234 nm as described in Section 3.4. Each substrate was prepared according to Chen and Whitaker [41].

### 3.9. Electrophoresis and Western Blotting

SDS-PAGE was performed in a Mini-PROTEAN^®^ 3 Cell electrophoresis apparatus (BIO-RAD, USA) according to the method of Laemmli [45] using 10% separating gel and 3.9% stacking gel. The gel were stained with PageBlue^TM^ Protein Staining Solution (Thermo Scientific, Waltham, MA, USA) with Coomassie Briliant Blue G 250 and destained in 25% ethanol, 10% acetic acid, and 65% redistilled water (*v*/*v*/*v*). As a standard, PageRuler^TM^ Plus Prestained Protein Ladder (Fermentas, Vilnius, Lithuania) within 11–250 kDa was used. Proteins separated by SDS-PAGE were transferred to a nitrocellulose membrane of 0.45 μm (Advantec^®^ MFS, Suite A Dublin, CA, USA) using TRANS-BLOT SD (BIO-RAD, Hercules, CA, USA) according to Towbin et al. [46]. As a primary antibody, polyclonal anti-LOX serum was prepared against the soybean LOX as described by Holková et al. [47]. Commercially available Anti-Rabbit IgG (H+L), HRP Conjugate (Promega, Madison, WI, USA) was used as a secondary antibody. The reaction was visualised with a TMB-stabilized (3,3′,5,5′-tetramethylbenzidine) substrate for horse radish peroxidase (Promega, Madison, WI, USA). The molecular mass of the purified enzyme was assessed by comparing the mobility of LOX protein with the mobility of molecular markers in 10% SDS-polyacrylamide gel. PageRuler^TM^ Plus Prestained Protein Ladder (11–250 kDa, Fermentas, Vilnius, Lithuania) was used to make a plot of the logarithm of molecular mass versus the relative mobility of protein bands.

## 4. Conclusions

In the present study, a novel LOX enzyme from California poppy seedlings was partially purified and biochemical properties of the enzyme were analyzed. The results indicate that the California poppy LOX belongs to nontraditional plant LOXs with dual positional specificity and has a relative molecular mass of 85 kDa. This dual positional specificity of the California poppy 9/13-LOX suggests that it is able to synthesize compounds that play a role in both the developmental process and defence response in plants. The optimal pH for LOX activity is 6.5, and the enzyme shows a high preference towards LA. Moreover, the results indicate that calcium may play an important role in regulating the activity of the LOX from California poppy. The K_M_ value of the LOX is 2.6 mM, and the V_max_ 3.14 μM/min/mg. The identification and characterization of plant LOX should prove to be a useful approach to interpret the physiological role of LOX in plants.

## Figures and Tables

**Table 1:** Purification of LOX from California poppy seedlings.

Purification Step	Proteins (mg/mL)	Activity (nkat/mL)	Specific Activity (nkat/mg)	Purification Fold
Crude extract	0.78	0.27	0.34	1
Phenyl-Sepharose CL-4B	1.07	0.48	0.49	1.4
Q-Sepharose	0.04	0.90	23.47	69
